# Avoiding overdiagnosis of parathyroid carcinoma

**DOI:** 10.1007/s00428-025-04274-4

**Published:** 2025-11-14

**Authors:** Carl Christofer Juhlin, Ozgur Mete

**Affiliations:** 1 Department of Oncology-Pathology, Karolinska Institutet, Stockholm, Sweden; 2 Department of Pathology and Cancer Diagnostics, Karolinska University Hospital, Stockholm, Sweden; 3 Department of Pathology, University Health Network, Toronto, ON Canada; 4 Endocrine Oncology Site, Princess Margaret Cancer Centre, Toronto, ON Canada; 5 Department of Laboratory Medicine and Pathobiology, University of Toronto, Toronto, ON Canada

**Keywords:** Parathyroid adenoma, Parathyroid carcinoma, Atypical parathyroid tumor, Overdiagnosis, Pathology

## Abstract

The diagnosis of a non-metastatic parathyroid carcinoma requires the demonstration of invasive growth that defines malignancy. These include angioinvasion or vascular invasion (i.e., tumor penetrating the vessel wall and associated with thrombus or intravascular tumor cells intermixed with thrombus), lymphatic invasion, perineural (intraneural) invasion, and/or direct invasion into adjacent anatomical structures. However, the distinction of a pT1 disease (8th edition of UICC TNM staging system) which represents a localized disease (tumor confined to the parathyroid gland or showing minimal extra-parathyroidal soft tissue invasion without direct invasion into adjacent structures) often requires meticulous microscopic examination that couples multiple levels and biomarker studies. Although the diagnostic criteria of malignancy are clearly defined, the identification of harbingers of invasive growth and distinguishing them from their mimics can pose diagnostic challenges. Several artifacts and manipulations can simulate malignancy. For example, a prior biopsy, PTH washout, ethanol injection, or any form of surgical manipulation may result in fibrosis, crush (mechanical) artifacts, or tissue distortion, which can obscure histological details and mimic invasion. Peliosis—the presence of extravasated erythrocytes without an endothelial lining—may simulate vascular invasion. Other common mimics include mechanically displaced intravascular tumor cells unassociated with thrombus, which can occur at the time of specimen handling. Extension of the tumor into the adjacent irregular connective tissue or pseudo-capsule can be mistaken for invasive growth. Similarly, an intrathyroidal location of the parathyroid gland adds another layer of complexity. In such cases, the boundary between the parathyroid tissue and surrounding thyroid parenchyma may not be clearly discernible, making it difficult to determine whether there is genuine invasive growth into thyroid (pT2 disease, 8th edition of UICC TNM system) or simply anatomical proximity. In addition, parathyromatosis and contour irregularities associated with long-standing secondary or tertiary hyperparathyroidism are other challenging manifestations. Atypical parathyroid tumors (WHO 2022) should also be clearly delineated using appropriate criteria. In summary, pathologists must be aware of the potential pitfalls that may lead to overdiagnosis of parathyroid carcinoma. A consolidated diagnostic workup, which combines multiple levels and biomarkers, is necessary to ensure diagnostic accuracy in all parathyroid tumors. This review provides practical insights on these diagnostic difficulties, illustrating common artifacts and mimics. We also discuss the relevant clinical, histological, immunohistochemical, and molecular features associated with parathyroid carcinoma, with the goal of enhancing diagnostic accuracy.

## Introduction

Parathyroid carcinoma (PC) is a rare endocrine malignancy, representing less than 1% of all cases of primary hyperparathyroidism (PHPT) [1–3]. Its estimated incidence is approximately 0.005 per 100,000 individuals annually. Unlike benign parathyroid disease, which predominantly affects women, PC shows a slight male predominance. The tumor most commonly presents in adults between the ages of 45 and 60, although it can occur across a wider age range [1, 4].

While the definitive diagnosis of PC is based on histopathological evaluation of the excised tumor, clinical suspicion may be raised preoperatively or intraoperatively based on a combination of clinical, biochemical, and surgical findings, as discussed in the following sections [3, 4]. Given the diagnostic complexity and the existence of benign mimics with overlapping features, it is essential to carefully evaluate all available data before confirming a diagnosis of carcinoma.

It is important to emphasize that although PC is, strictly speaking, a histological diagnosis, it is rarely made in isolation. Instead, it relies on an integrative analysis that includes clinical context, biochemical profile, imaging, intraoperative observations, and ancillary testing. For instance, a small parathyroid lesion in a patient with only mildly elevated calcium and parathyroid hormone (PTH) levels is more likely to represent a parathyroid adenoma [3]. In such cases, findings like questionable vascular invasion or subtle tumor border irregularities should be interpreted with caution to avoid overdiagnosis.

In the sections that follow, we will review the well-established histopathological, immunohistochemical, and molecular characteristics associated with parathyroid adenoma, parathyroid carcinoma, and atypical parathyroid tumor. We will also highlight common diagnostic pitfalls and artifacts that may lead to misclassification, with the aim of promoting accurate and judicious diagnosis in this challenging area of endocrine pathology.

## Parathyroid adenoma

From a clinical perspective, there is a clear-cut correlation between parathyroid adenoma size and biochemical severity in primary hyperparathyroidism. Smaller adenomas, typically weighing 40–100 mg, are often associated with mild or asymptomatic disease and exhibit normal to mildly elevated serum calcium and parathyroid hormone (PTH) levels [5, 6]. In contrast, larger adenomas—particularly those exceeding 1 cm—are more frequently linked to higher calcium and PTH concentrations and a greater risk of skeletal involvement or, in rare cases, hypercalcemic crisis [7]. Although uncommon, “giant” parathyroid adenomas can develop, sometimes measuring several centimeters in greatest dimension, and may present with dramatic clinical manifestations.

Histologically, parathyroid adenomas are typically well-circumscribed lesions composed of uniform chief cells, transitional cells, oncocytic cells, or water-clear cells [3]. In more than half of cases, a rim of residual non-tumorous parathyroid parenchyma (often referred as to “atrophic rim”) can be identified at the periphery of the lesion provided the parathyroid gland is grossly sectioned horizontally through the vascular hilum [8]. A hallmark of conventional parathyroid adenomas is the marked reduction or absence of stromal fat, which contrasts with normal parathyroid architecture. Chief cells generally have small, round nuclei with minimal cytologic atypia, no prominent nucleoli, and scant cytoplasm. The overall cytologic blandness, well-defined borders, and absence of high-risk features usually make the diagnosis of adenoma straightforward [3, 9].

Immunohistochemically, parathyroid adenomas typically show low proliferative activity, reflected by a low Ki-67 labeling index (less than 5%) and ≤ 5 mitoses per 10 mm^2^ [3, 8, 10–14]. Retention of parafibromin (biomarker discussed in detail in later sections), APC, and Bcl2 expression is the norm, while markers such as Galectin-3 and PGP9.5 are typically negative—findings that may help distinguish adenomas from atypical parathyroid tumors, an entity with an acknowledged risk of future recurrences [3]. Although rare histologic subtypes exist, including water-clear cell adenomas and lipoadenomas, these do not pose diagnostic challenges with respect to malignancy [3, 15–18]. However, their recognition in the context of primary hyperparathyroidism is important [18]. Nonetheless, the tumor cells in these subtypes retain immunoreactivity for GATA3 and parathyroid hormone (PTH), which reliably confirms their parathyroid origin and supports the diagnosis [3, 10, 19]. Similar to C-cell neoplasia and other neuroendocrine neoplasms, it is important to emphasize that monoclonal PAX8 (C terminus specific) is not expressed in parathyroids and their tumors; however, polyclonal PAX8 antibodies can stain parathyroid tumors [10, 20]. Therefore, pathologists are required to know and document the clone of PAX8 used when reviewing their PAX8 assay.

## Parathyroid carcinoma

Parathyroid carcinoma (PC) is a rare endocrine malignancy that often presents diagnostic challenges, particularly in the absence of overt (gross)invasion or metastasis [3]. Clinical suspicion typically arises from a constellation of features rather than a single defining sign. A firm, palpable cervical mass and a visibly enlarged parathyroid gland—especially those exceeding 3 cm—should raise concern [1, 4, 14, 21]. Biochemical indicators such as marked hypercalcemia, with serum calcium levels surpassing 3.0 mmol/L (or 12 mg/dL), are more frequently associated with malignancy than with benign parathyroid disease. These findings are often accompanied by strikingly elevated parathyroid hormone (PTH) levels, frequently several times above the normal upper limit [4, 14, 21]. Intraoperative findings such as firm adhesions or apparent invasion into adjacent structures further contribute to clinical suspicion. Although none of these features is individually diagnostic, their combination should prompt heightened vigilance and guide for a more detailed pathological assessment.

Parathyroid carcinoma is overrepresented in certain hereditary syndromes, most notably hyperparathyroidism-jaw tumor (HPT-JT) syndrome, which is caused by pathogenic germline *CDC73* variants [22–24]. Individuals with HPT-JT are at significantly higher risk of developing PC compared to those with other hereditary conditions such as multiple endocrine neoplasia type 1 (MEN1) or familial isolated hyperparathyroidism (FIHP), in which the overall risk remains low [9]. *CDC73* encodes parafibromin, a nuclear protein involved in transcriptional regulation and tumor suppression [22, 25–27]. Germline mutations typically lead to loss of function, and most pathogenic variants either introduce premature stop codons or affect key regulatory domains essential for nuclear localization. As a result, mutated parafibromin often fails to accumulate in the nucleus, which can be demonstrated by immunohistochemistry [13, 28–31].

**Fig. 1:**
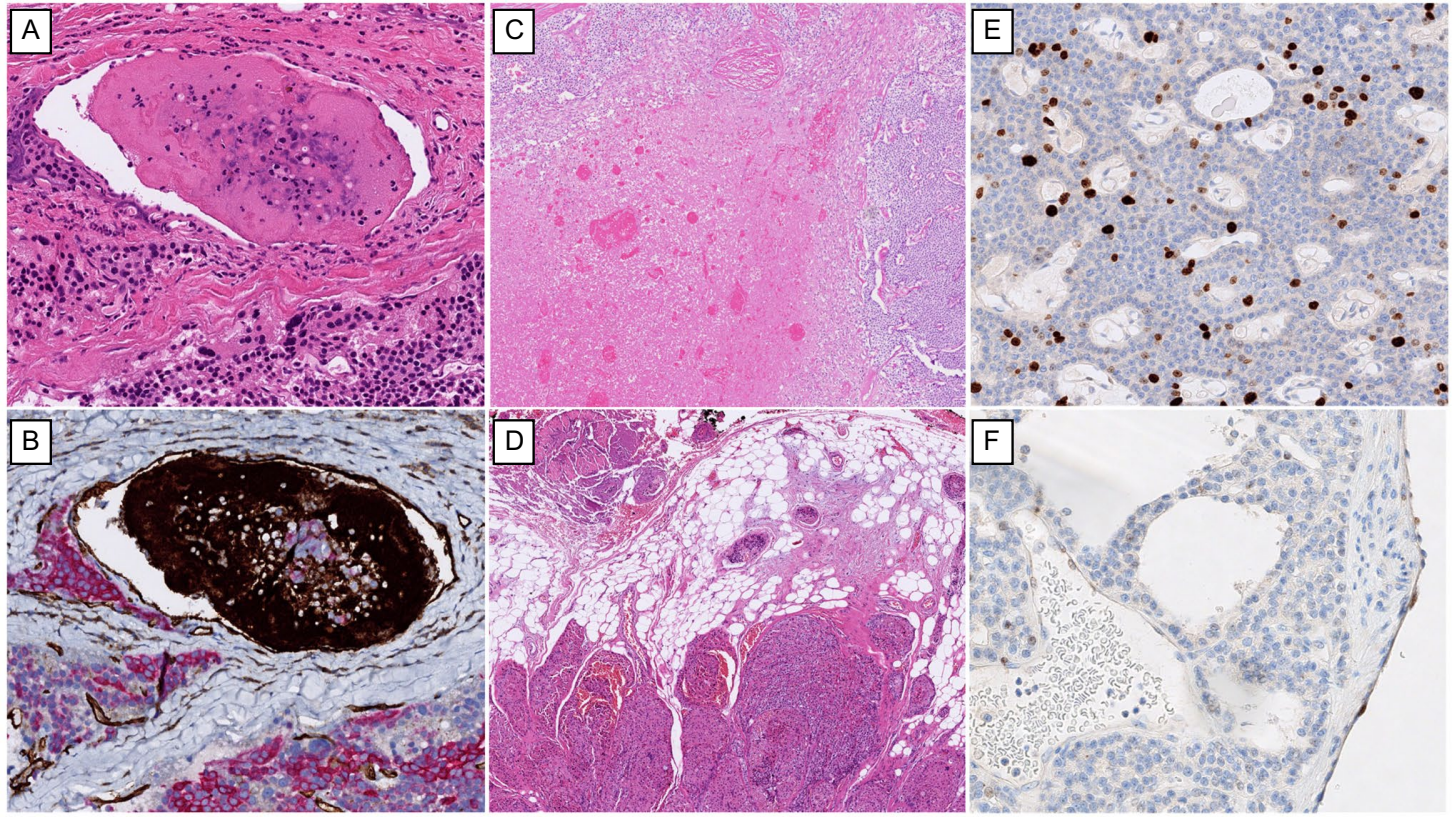
Histological and immunohistochemical features of parathyroid carcinoma. **A** True vascular invasion is a hallmark of parathyroid carcinoma. Note the associated fibrin (platelet) clot and sparsely distributed intravascular tumor cells. **B** The intravascular location of tumor cells is confirmed by CD31 immunohistochemistry (brown) highlighting the endothelium, and pan-cytokeratin staining (red) marking epithelial tumor cells. In this case, CD31 also stains fibrin tissue reaction since CD31 can be identified in platelets as well as in other inflammatory cells. However, CD61 (not illustrated herein) is more commonly used to highlight platelets in surgical pathology. **C** Geographic (coagulative) necrosis is rare in parathyroid adenomas and should raise suspicion for carcinoma. **D** Vascular invasion (in the adipose tissue) and infiltrative growth into adjacent adipose tissue in parathyroid carcinoma. **E** The Ki-67 labeling index typically exceeds 5%; in this tumor, the index approaches 10%, supporting a diagnosis of carcinoma. **F** Loss of nuclear parafibromin is a recurring finding in parathyroid carcinoma and is highly suggestive of an underlying *CDC73* mutation. Note the retained nuclear staining in non-tumorous endothelial cells (right), serving as an internal control

Parafibromin immunohistochemistry has become a valuable diagnostic tool in the evaluation of parathyroid tumors [3, 9, 10]. In the setting of primary hyperparathyroidism, loss of nuclear parafibromin staining strongly suggests an underlying *CDC73* mutation and supports a diagnosis of malignancy [28, 30, 31] (Fig. 1). Although most sporadic parathyroid adenomas retain parafibromin expression, reduced or absent nuclear staining is commonly observed in parathyroid carcinomas. Interestingly, atypical staining patterns—such as focal nuclear loss or selective nucleolar loss—have also been described in tumors with specific *CDC73* mutations that may alter the protein’s nuclear or nucleolar localization [31, 32]. Despite some variability related to staining protocols and antibody selection, parafibromin immunohistochemistry remains a highly informative adjunct, especially when interpreted alongside internal controls such as vascular endothelial cells. Diffuse nuclear loss is particularly meaningful and should also prompt consideration for germline *CDC73* sequencing, especially when clinical or histological features are suspicious for carcinoma, as well as in any patients ≤ 45 years old with primary hyperparathyroidism [3, 9]. Interestingly, morphologic hallmarks of parafibromin-deficient parathyroid tumors include eosinophilic cells with a perinuclear halo, sheet-like growth, coarse chromatin, and arborizing vasculature [33, 34]. These features may help triage cases for parafibromin immunohistochemistry. Parafibromin deficiency alone does not warrant a diagnosis of malignancy since germline or somatic *CDC73*-driven parathyroid adenomas can also feature parafibromin deficiency [34, 35]. Atypical parathyroid tumors with parafibromin deficiency are considered at high risk of disease recurrence [3].

Histologically, parathyroid carcinomas tend to be larger and heavier than adenomas and often display prominent fibrosis and irregular borders [3]. Common microscopic features include broad fibrous bands, coagulative tumor necrosis, a solid growth pattern, and increased mitotic activity [1, 3, 9, 14, 36] (Fig. 1). Prominent macronucleoli may also be present. However, these features alone are not diagnostic, and similar changes can occur in benign conditions such as secondary hyperparathyroidism or in lesions previously subjected to fine-needle aspiration or ethanol ablation [8, 14]. The identification of coagulative tumor necrosis or atypical mitoses is very concerning (but not diagnostic of malignancy) in the absence of a former manipulation to the gland [3]. In such cases, multiple levels can facilitate the search of microscopic invasive growth (e.g., vascular invasion, perineural invasion) to distinguish an atypical parathyroid tumor from a parathyroid carcinoma. A diagnosis of carcinoma should therefore rest on definitive evidence of invasive behavior, such as vascular, lymphatic, or perineural invasion, direct extension into surrounding structures (including peri-parathyroidal soft tissue), or the presence of metastatic disease [3] (Fig. 1). It is important to emphasize that vascular invasion should be searched at the intersection of the tumor and non-tumorous parenchyma as well as beyond the tumor periphery. A proposed histological triad—including coagulative necrosis, macronucleoli, and a mitotic rate exceeding 5 per 10 mm^2^ (originally five per fifty high-power fields)—may serve as a helpful prompt but lacks absolute specificity [36]. Reticulin histochemistry has been proposed as a method to detect disrupted reticulin fibers, which may correlate with biologically aggressive parathyroid tumors based on limited data; however, this finding requires further validation for routine diagnostic use [37, 38].

Among the earliest biomarkers proposed for parathyroid carcinoma was Ki-67 (MIB1), a proliferation marker that highlights cells in active phases of the cell cycle. While carcinomas generally show higher Ki-67 indices than adenomas, there is significant overlap, limiting its diagnostic utility as a standalone marker [3, 11, 12, 19, 39]. However, when used in conjunction with other markers, Ki-67 can provide useful supportive evidence. More diagnostically informative are immunohistochemical panels incorporating loss of parafibromin, RB, p27, BCL2, MDM2, and APC expression, alongside overexpression of galectin-3, PGP9.5, and p53 [3, 10, 12, 19, 29, 40–46]. A Ki-67 index greater than 5% further strengthens the case for malignancy, particularly when paired with loss of cell cycle regulatory proteins [3] (Fig. 1).

Recent advances in genomic and epigenomic profiling are gradually deepening our understanding of PC pathogenesis. Somatic *CDC73* mutations, which are present in a substantial proportion of sporadic carcinomas, are associated with increased copy number variation, a higher overall mutational burden, and poorer clinical outcomes [9, 47]. Next-generation sequencing studies have also identified recurrent alterations in genes such as *ADCK1*, *PRUNE2*, and *KDR*, as well as altered PI3K/AKT/mTOR and Wnt pathways, and gene alterations involved in DNA repair, chromatin remodeling, DNA and histone methylation, cell cycle regulation, and regulators of angiogenesis [48–51]. Isolated mutations have been reported in canonical cancer drivers including *SDHA* and *DICER1* [50]. It is worth documenting that actionable molecular alterations were identified in around 54% of advanced parathyroid carcinomas in a series from the MD Anderson Cancer Centre [50]. Therefore, the role of actionable biomarker testing algorithms similar to thyroid carcinomas [52] may be implemented in the clinical management of parathyroid carcinomas, particularly those lacking parafibromin deficiency.

Although the data are scant, the molecular landscape of parathyroid adenomas identified in longstanding secondary or tertiary hyperparathyroidism seems to deviate from primary hyperparathyroidism-related parathyroid adenomas [51]. The molecular data on rare parathyroid carcinomas arising in the background of tertiary hyperparathyroidism are largely unknown.

Mutations in the *TERT* promoter, a common mechanism of telomerase activation in many cancers, appear to be rare in parathyroid carcinoma despite reports of TERT protein overexpression [53, 54]. This discrepancy points to alternative, as yet undefined, mechanisms driving telomerase activity in these tumors. Epigenetic disruption is also emerging as a key factor. Genome-wide methylation analyses have shown that parathyroid carcinomas exhibit hypermethylation of several tumor suppressor genes, including *CDKN2A*, *CDKN2B*, *WT1*, and multiple members of the SFRP family [55]. Recurrent methylation of the *CDC73* and *APC* promoter regions has also been documented [43, 56]. In the case of *APC*, methylation may account for protein loss in the absence of gene mutations, potentially contributing to aberrant activation of Wnt signaling pathways. More broadly, parathyroid carcinomas display reduced levels of 5-hydroxymethylcytosine and diminished expression of the TET1 demethylase enzyme, suggesting a global defect in DNA demethylation [57].

While next-generation sequencing and methylation profiling hold promise for improved diagnosis and therapeutic targeting, these technologies are not yet widely available in routine clinical practice. At present, immunohistochemistry remains the most practical and informative adjunct to histological evaluation. In particular, parafibromin expression serves as a critical marker, offering diagnostic, prognostic, and genetic insights in a single assay. When interpreted in the context of clinical findings, histology, and ancillary testing, these tools collectively enhance the diagnostic precision and management of this rare but clinically important malignancy.

## Atypical parathyroid tumor

**Fig. 2:**
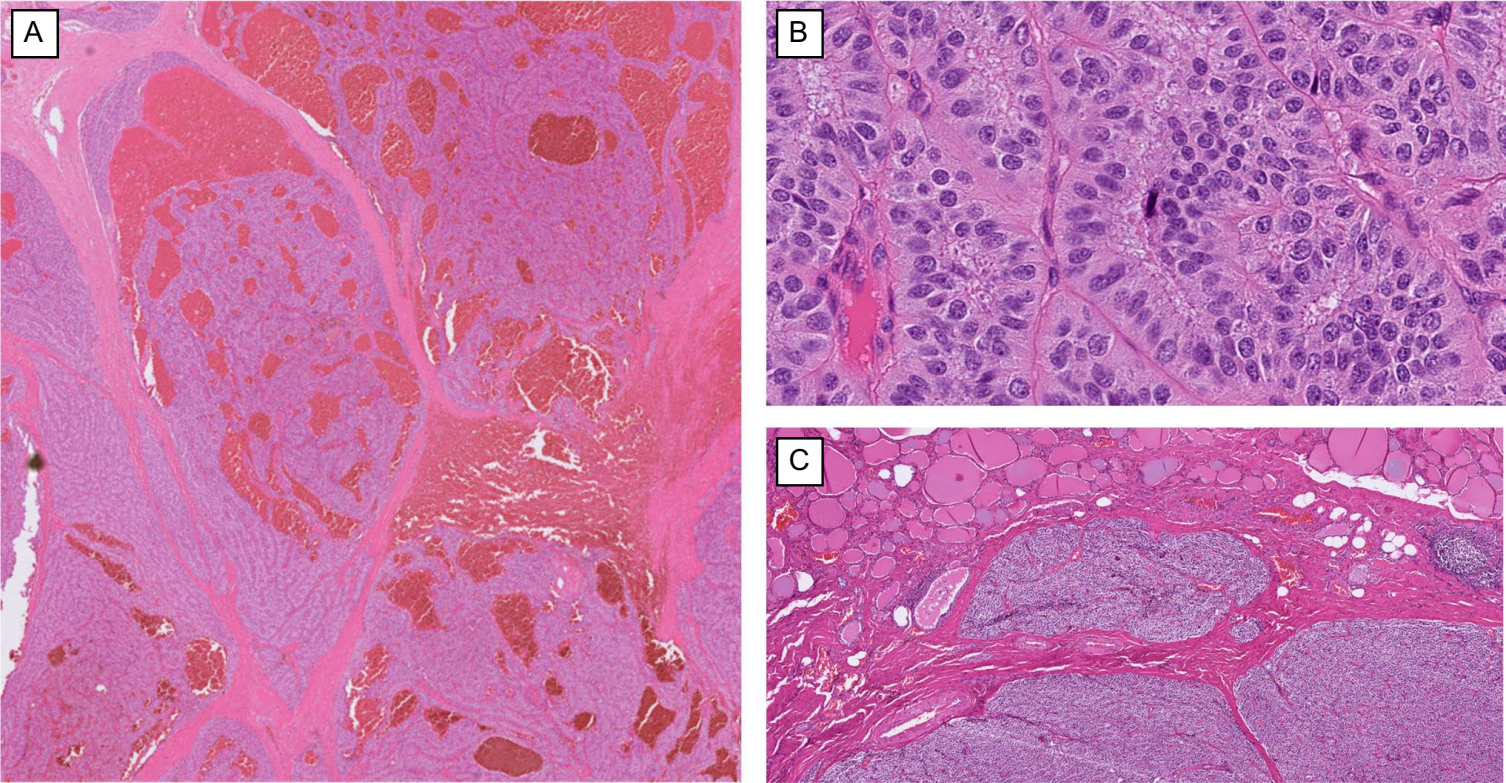
Histological features of an atypical parathyroid tumor. **A** Broad fibrous bands intersect this large, atypical parathyroid tumor, making it difficult to distinguish the true tumor capsule from internal fibrous septa. **B** In primary hyperparathyroidism, increased mitotic count (> 5 mitoses per 10 mm^2^) is uncommon in parathyroid adenomas and indicates an atypical parathyroid tumor for a non-invasive parathyroid tumor in the absence of a former manipulation. **C** An intrathyroidal parathyroid tumor exhibiting an ambiguous interface with adjacent thyroid parenchyma, complicating the assessment of true invasion since parathyroids do not have true capsule

The diagnostic category of “atypical parathyroid tumor” is a distinct non-invasive parathyroid tumor that displays worrisome histological features yet falls short of the unequivocal criteria required for a diagnosis of parathyroid carcinoma [3, 9]. These neoplasms typically show thick fibrous bands, a trabecular or solid architecture, focal capsular entrapment of tumor nests, increased mitotic or proliferative activity, atypical mitosis, necrosis, and conspicuous nuclear atypia with macronucleoli [3, 36, 58] (Fig. 2). What they lack, by definition, is hard evidence of malignancy: there is no verified vascular, lymphatic, or perineural invasion, no direct extension into surrounding soft tissues or thyroid parenchyma, and no regional or distant metastasis.

In the past, many of these lesions were labeled “atypical parathyroid adenoma,” but that designation largely disappeared as ancillary studies and stricter diagnostic thresholds have improved classification. Careful correlation with clinical context—such as preoperative cytology, multiglandular disease, multiple endocrine neoplasia type 1, or prior medical manipulation—often explains the reactive fibrosis or mitotic figures that first raised concern. When morphology remains ambiguous, a rigorous diagnostic workup that combines additional histologic levels (to hunt for invasion) and biomarker studies to search for pathologic aberrancies involving parafibromin, RB, p27, APC, galectin-3, and Ki-67 provides additional guidance [10, 19, 28, 31, 31, 45, 59–61]. For example, diffuse loss of parafibromin or APC, and PGP9.5 reactivity heightens the suspicion of malignancy, whereas intact staining profiles for parafibromin or APC, and lack of PGP9.5 reactivity may support a benign process. Recent molecular studies echo this stratification, showing that a minority of atypical tumors harbor *CDC73* or other high-risk genetic lesions and that those cases are the ones most likely to behave aggressively [62, 63].

Most atypical parathyroid tumors follow an indolent course after complete excision, but a small subset later recurs or metastasizes, underscoring the need for vigilant follow-up [3, 62]. Therefore, these tumors are considered parathyroid tumors of uncertain to low-malignant potential. Until molecular markers become part of routine practice, the current approach combines meticulous histologic assessment, targeted immunohistochemical biomarker testing, and informed clinical correlation to identify the few lesions that carry genuine malignant potential while sparing the majority from overtreatment or unnecessary follow-up.

## Diagnostic dilemmas and mimics of invasion

Several preoperative and intraoperative factors can induce histological changes that mimic true invasion, and it is imperative for the endocrine pathologist to recognize these risks to avoid overdiagnosing parathyroid carcinoma. The following sections explore various types of artefacts, with a focus on their potential to lead to diagnostic misinterpretation.

### Preoperative fine needle aspiration biopsy artifacts

**Fig. 3:**
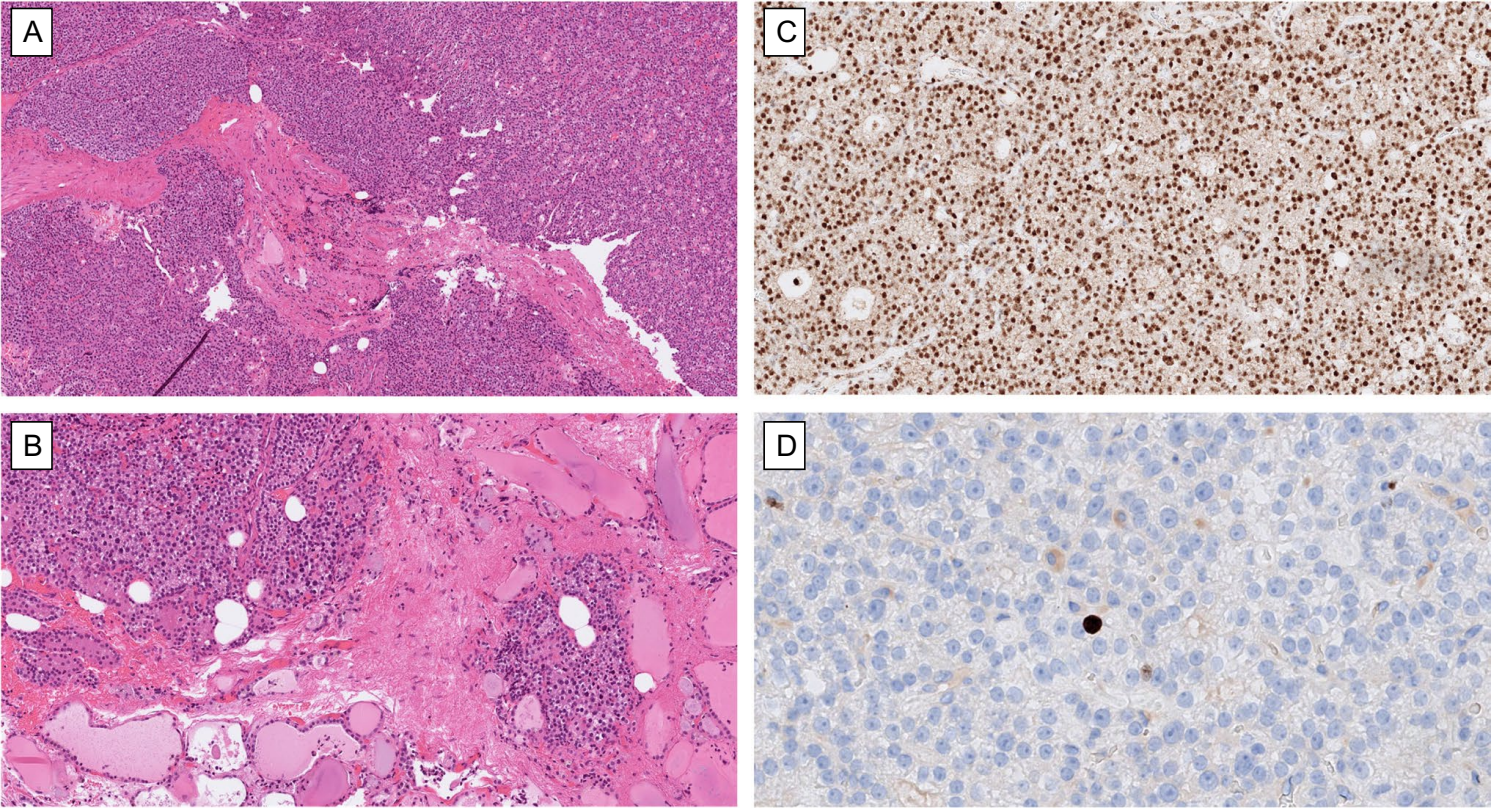
Histologic pitfalls in diagnosing parathyroid tumors: Post-FNA changes in an intrathyroidal parathyroid gland. **A** Intrathyroidal parathyroid gland previously subjected to fine-needle aspiration (FNA), followed by surgical excision. Histological examination reveals irregular fibrosis and sparse inflammatory infiltrates, including pigment-laden macrophages along the needle tract. These findings should not be mistaken for true fibrous bands or capsular invasion. **B** The same tumor shows displaced parathyroid cells within fibrotic stroma, resulting in an equivocal interface with adjacent thyroid parenchyma. This must not be overinterpreted as true invasive growth. **C** Widespread retention of nuclear parafibromin immunoreactivity supports a benign diagnosis. **D** The Ki-67 labeling index was well below 1%, further suggesting an indolent, non-malignant nature

A prior history of manipulation to the parathyroid glands—such as fine-needle aspiration biopsy (FNAB), PTH washout, or ethanol injection—is critical information that must be communicated to the pathologist when assessing parathyroid lesions [3, 8]. These procedures can induce histological changes like capsular defects, fibrosis, and other tissue disruptions that may closely resemble true invasive features [64–66] (Fig. 3). Without this context, there is a significant risk of misinterpreting such artefacts as signs of malignancy. Studies have shown that biopsy-related artefacts are common in parathyroid tumors that underwent FNAB before excision, underscoring the importance of accurate clinical-pathological correlation to avoid diagnostic errors [65].

### Mechanical artefacts

**Fig. 4:**
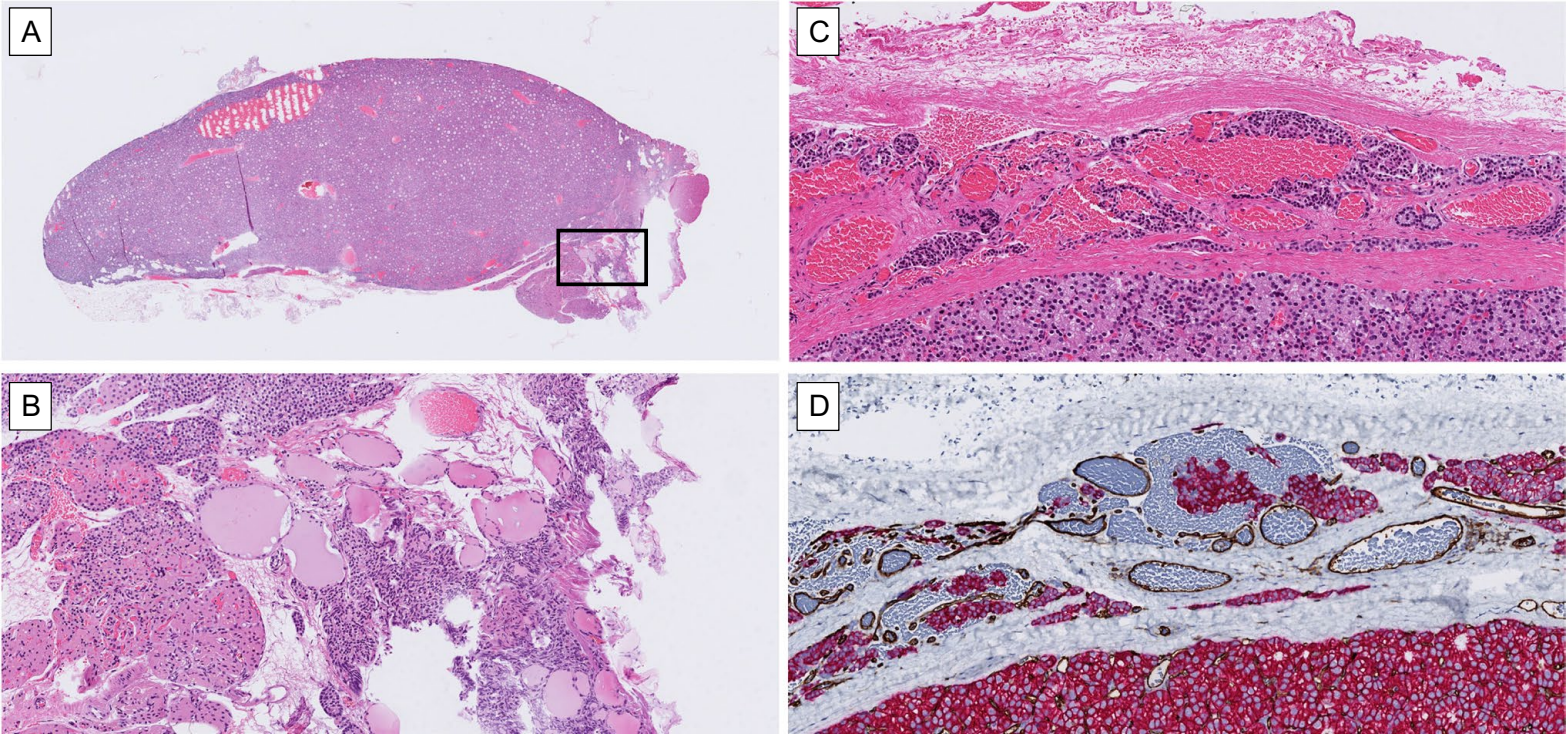
Histologic pitfalls mimicking invasion in parathyroid adenomas: Mechanical artefacts and peliosis. **A**, **B** A small parathyroid adenoma lacking worrisome features may still present diagnostic challenges due to crush artefacts in the adjacent rim of normal parathyroid tissue. These artefacts can distort the tissue architecture and create the illusion of tumor infiltration into adipose or thyroid tissue (detail magnified in **B**). **C** Peliosis, characterized by lakes of extravasated erythrocytes without endothelial lining, can mimic vascular invasion—particularly when interspersed with parathyroid cells. **D** Double immunohistochemistry for CD31 (brown) and pan-cytokeratin (red) helps confirm the absence of true endothelial-lined vascular channels, distinguishing peliosis from genuine vascular invasion

Parathyroid surgery, while typically precise, can inadvertently introduce artefacts due to digital manipulation or the use of forceps during gland extraction. These mechanical stresses may result in tissue deformation or “crush artefacts,” which can also lead to displacement of tumor cells in a way that mimics vascular or capsular invasion. In particular, intraluminal tumor cell deposits that lack a clear, continuous growth pattern from the main tumor into the vessel should raise suspicion for contamination—especially in the absence of a reactive fibrinoid response. The use of CD61 immunohistochemistry can support the assessment of fibrin/platelet response, which is typically present in vascular invasion. Moreover, even a crushed rim of normal parathyroid tissue in the adjacent fat may be hard to tell apart from tumor cells (Fig. 4). Recognizing such artefacts is essential to avoid overdiagnosis of malignancy based on surgical or handling-related changes.

### Peliosis

Peliosis is a histological phenomenon characterized by the presence of multiple blood-filled spaces within the parenchyma of an organ. These spaces lack an endothelial lining, distinguishing peliosis from true vascular structures. While most commonly observed in the liver (peliosis hepatis), it can also occur in the spleen, bone marrow, and endocrine and neuroendocrine organs (e.g., non-tumorous pancreatic islets) and their tumors, including those arising in the parathyroid gland [67, 68]. In parathyroid tumors, peliosis is not uncommonly observed both at the periphery and within central areas of the lesion. When located near the capsule and adjacent to tumor cells, peliosis may mimic vascular invasion (Fig. 4). Immunohistochemistry for endothelial markers such as CD31 and ERG is particularly useful in these cases, as the absence of an endothelial lining in peliosis helps differentiate it from true vascular structures, aiding in accurate diagnosis (Fig. 4).

### Intrathyroidal parathyroid glands

Intrathyroidal parathyroid glands account for approximately 2% of primary hyperparathyroidism cases [69]. In parathyroid carcinoma staging, direct invasion into the thyroid gland warrants pT2 disease (8th edition of the UICC TNM staging scheme). Intrathyroidal parathyroid tumors can pose a significant diagnostic challenge for multiple reasons. First of all, these tumors may be mistaken for a primary thyroid nodule and may have been subjected to a former FNAB. As a consequence, the expansile (non-infiltrative) growth of parathyroid tumors in the thyroid pseudo-capsule or in the thyroid parenchyma may mimic invasive growth in the setting of post-biopsy induced fibrosis and irregular borders (Fig. 3). In such cases, the distinction between a truly invasive parathyroid carcinoma and a well-demarcated intrathyroidal parathyroid neoplasm respecting anatomical boundaries may become difficult. The identification of vascular invasion or perineural invasion would warrant malignancy. Biomarker aberrancies should also alert the pathologist. Therefore, additional levels and biomarker studies should be coupled with careful assessment of the interface, attention to the presence of intervening fibrous tissue, and correlation with gross findings and clinical context.

Most parathyroid tumors are unencapsulated neoplasms. Even in the exceptional examples of encapsulated parathyroid tumors, tumor extension into its capsule does not warrant malignancy since the invasive growth that defines malignancy in parathyroid tumors requires direct invasion into adjacent structures. Therefore, an analogy with encapsulated follicular cell-derived thyroid tumors is not applicable to parathyroid tumors.

### Parathyromatosis

Parathyromatosis refers to the presence of multiple microscopic foci of parathyroid tissue scattered in soft tissue, most commonly in the neck or mediastinum, and is typically encountered in patients with a history of parathyroid surgery or needle biopsy [70]. It is believed to result from mechanical disruption of the parathyroid gland during surgical manipulation, biopsy, or rupture of a cystic lesion, leading to inadvertent seeding or implantation of parathyroid cells into surrounding tissues [8]. Histologically, these foci may mimic invasive or metastatic deposits of parathyroid carcinoma, particularly when they appear embedded within adipose tissue or a fibrous stroma. However, unlike parathyroid carcinoma, parathyromatosis usually lacks cytological atypia, increased mitotic activity or atypical mitosis, or a desmoplastic stromal response, and the parathyroid tissue islands are often small, uniform, and well-circumscribed. Recognition of this entity is critical, as misinterpretation can lead to an incorrect diagnosis of malignancy and unnecessary aggressive treatment. Clinical history, particularly former surgical or procedural intervention, is essential in making this distinction. A recent study aimed at distinguishing parathyromatosis and parathyroid carcinoma found that parathyroid carcinoma showed higher rates of coarse chromatin, infiltrative invasive growth, and metastasis, while parathyromatosis uniquely exhibited “circumscribed or expansile growth” a phenomenon defined by the authors as multiple well-circumscribed nests of tumor cells present in soft tissue, without destruction of existing tissue or desmoplastic response [71].

### Changes associated with secondary hyperparathyroidism and other rare conditions

**Fig. 5:**
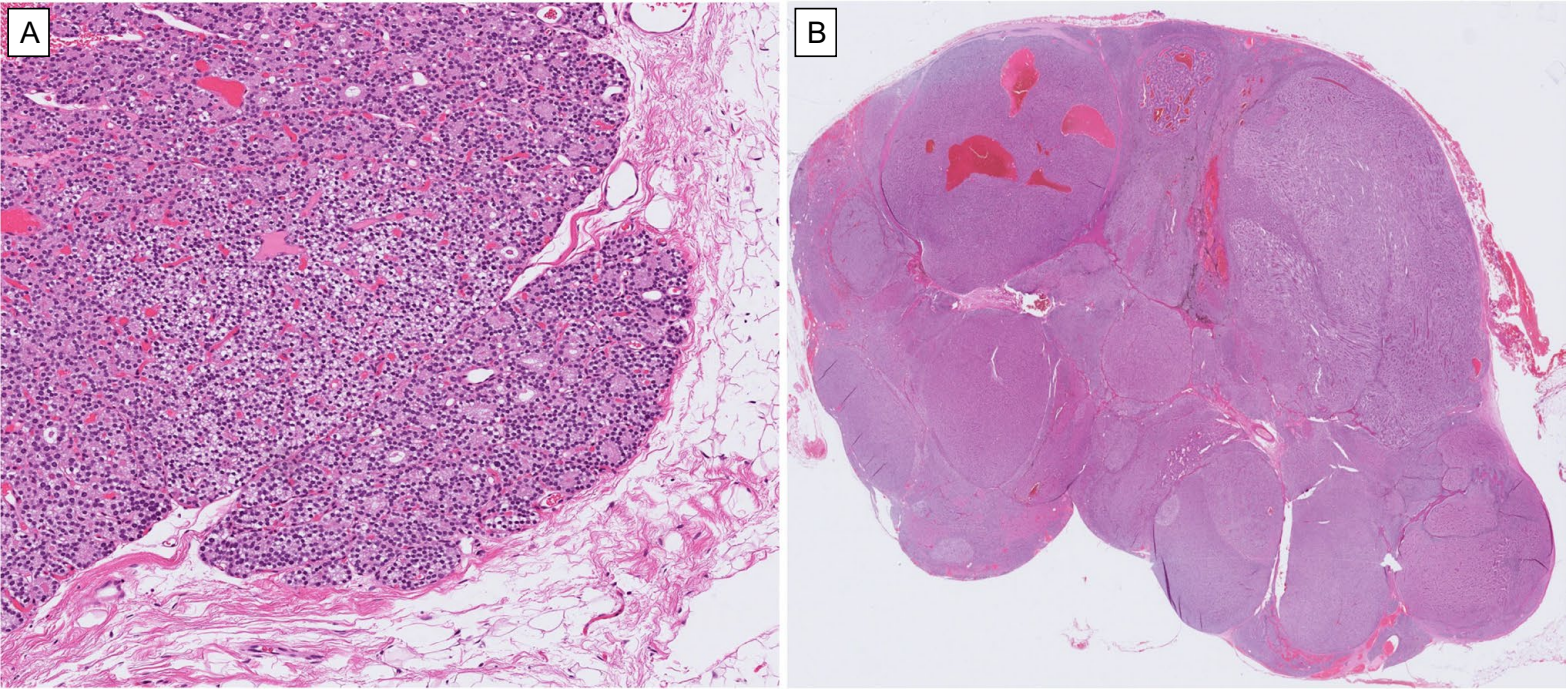
Pseudoinvasion in secondary hyperparathyroidism: architectural mimics of carcinoma. **A** A mushroom-like protrusion of parathyroid tissue through the capsule may resemble invasive growth but is not, by itself, diagnostic of carcinoma. Such features, as seen in this case of secondary hyperparathyroidism, should be interpreted with caution and in the context of other findings. **B** Broad fibrous bands in secondary hyperparathyroidism dividing lobules of chief cells may mimic invasive nodules; however, these architectural features alone do not confirm malignancy and should not be overcalled as carcinoma without definitive invasive criteria

Parathyroid glands affected by chronic secondary hyperparathyroidism often exhibit pronounced nodular hyperplasia with irregular glandular contours, which can closely mimic features typically associated with malignancy [3]. These contour irregularities, resulting from the expansion of hyperplastic nodules and fibrous remodeling, may resemble capsular irregularity or even extracapsular extension under the microscope (Fig. 5). In some cases, nodules may appear to bulge into or through a thickened capsule, raising concern for invasion (Fig. 5). Additionally, longstanding proliferative stimulation from diminished vitamin D levels due to chronic kidney disease may lead to increased mitotic count (> 5 mitoses per 10 mm^2^) and fibrosis, both features of which further complicate the histological distinction from parathyroid carcinoma [72]. Similar findings can be identified in a subset of primary hyperparathyroidism-related multiglandular parathyroid adenomas in MEN1 syndrome, as well as in the setting of chronic lithium administration [8]. It is therefore essential for the pathologist to interpret such findings in the context of the patient’s clinical history, including the presence of renal disease or other causes, and to apply strict diagnostic criteria to avoid overcalling malignancy in a reactive or hyperplastic setting.

## Comprehensive algorithm to assess parathyroid tumors with  worrisome findings

Parathyroid tumors with worrisome features should raise suspicion for parathyroid carcinoma and warrant careful evaluation by a pathologist with specific expertise in parathyroid neoplasia. As a first step, consultation with an experienced colleague is strongly recommended in cases with ambiguous or borderline findings. Accurate diagnosis relies on an integrated approach that combines clinical presentation, perioperative impressions, histological architecture, immunohistochemical profiles, and, in selected cases, molecular data. It is essential to confirm that the entire lesion has been submitted for histological examination, as additional tissue levels may reveal microscopic (often single focus of) vascular invasion or other diagnostic criteria not apparent on initial sections.

**Fig. 6:**
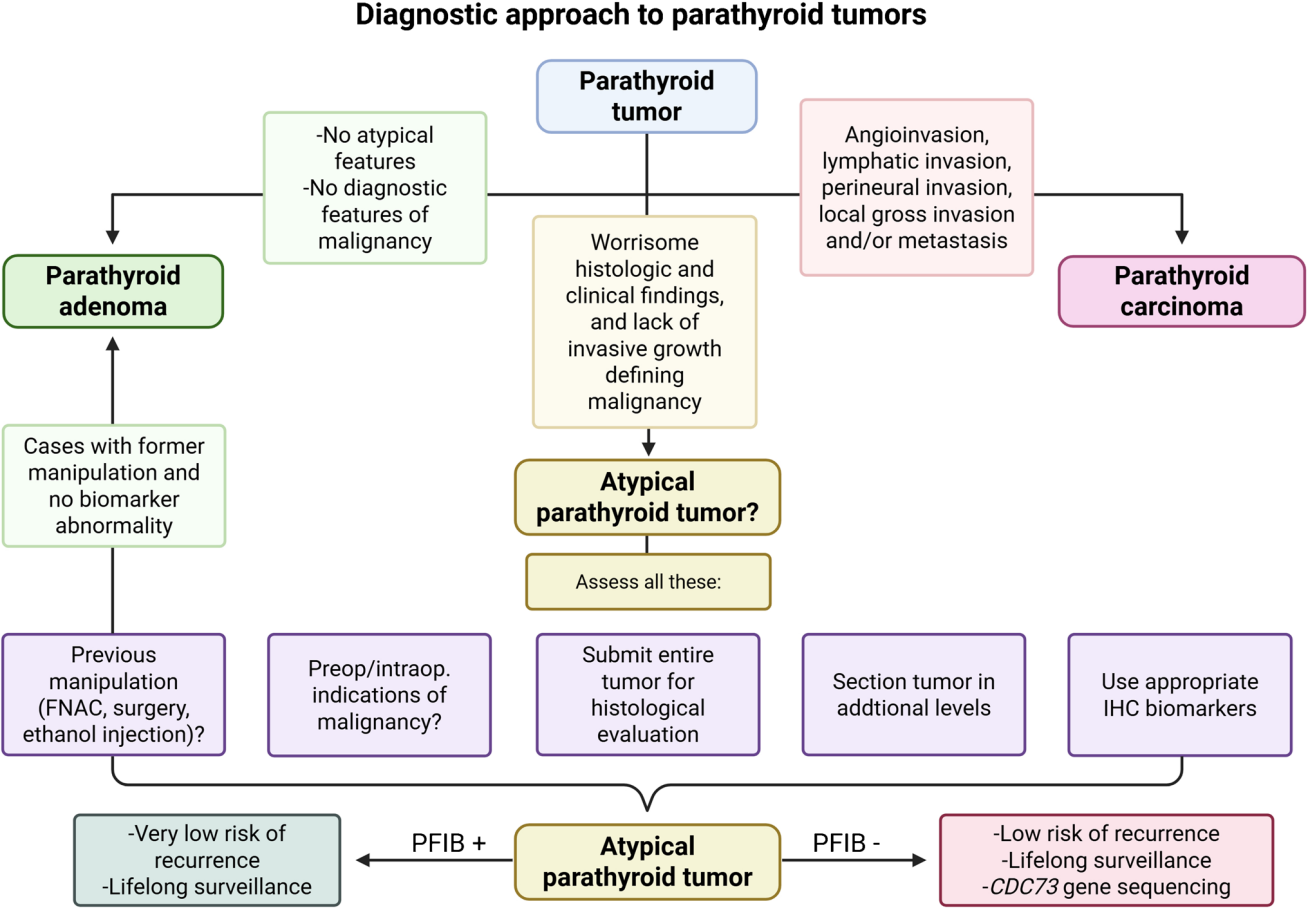
Schematic overview of the diagnostic approach to parathyroid tumors. IHC, immunohistochemistry; FNAC, fine needle aspiration cytology; PFIB, parafibromin immunohistochemistry. Created using BioRender.com

Given the diagnostic complexity, meticulous attention to detail is essential. Not all centers have access to advanced immunohistochemical markers or molecular assays, and pathologists should be prepared to refer challenging cases for external consultation when needed. A summarized diagnostic approach for evaluating parathyroid tumors that cannot be readily classified as adenomas is provided in Table 1. Moreover, a schematic overview of the diagnostic approach to parathyroid tumors is presented in Fig. 6.

**Table 1:** Assessment and pitfalls of suspicious parathyroid tumors

Assessment step	Key clues of carcinoma	Common pitfalls	Interpretation
Clinical	Severe hypercalcemia, markedly elevated PTH, gland > 3 cm, palpable mass, HPT-JT history, metastatic disease	Mild biochemistry with atypical histology	Strong biochemical and clinical findings raise suspicion for carcinoma; only metastatic disease is diagnostic of carcinoma (but histological confirmation of the parathyroid origin of a metastatic disease is often required)
Intraoperative	Direct gross invasion into organs/structures	Tissue distortion from manipulation	Use gross findings as supportive, not definitive
Histology	Diagnostic: Vascular invasion, lymphatic invasion, perineural invasion, or invasion into surrounding organs, or histologically confirmed metastasis Non-specific frequent findings: fibrous bands, increased mitotic count (> 5 mitoses per 10 mm^2^), atypical mitotic figures, coagulative tumor necrosis	Artefact from FNAB, mechanical artifacts, peliosis, intrathyroidal location, parathyromatosis, secondary HPT, chronic lithium administration	Diagnose carcinoma only with unequivocal (diagnostic) invasive growth or metastatic disease; otherwise consider atypical parathyroid tumor
Immunohistochemistry	Loss of parafibromin, RB, APC; high Ki-67; positivity for PGP9.5, and other biomarker aberrancies	Misinterpretation due to weak or very focal staining; lack of controls	Parafibromin loss is a strong marker for malignancy and *CDC73* mutation; parafibromin deficiency should trigger germline *CDC73* testing
Molecular	*CDC73* mutation (germline/somatic), NGS in individual cases	Limited access or inconclusive results	Supportive in difficult cases, especially with parafibromin loss and when actionable molecular alterations are needed to treat advanced parathyroid carcinomas
Final Diagnosis	Integration of all above	Overcalling endocrine atypia as malignancy	Only call carcinoma with definitive invasion (most reliable criterion is vascular invasion); label as atypical tumor if features are suspicious but not diagnostic

## Concluding remarks

Diagnosing parathyroid carcinoma remains one of the most challenging areas in endocrine pathology due to its rarity, histological overlap with benign lesions, and the often subtle or equivocal nature of its defining features. While a definitive diagnosis relies on histological evidence of invasive behavior—such as vascular or perineural invasion, invasion of adjacent organs, or metastasis—such features are not always overtly present. Consequently, the pathologist is frequently faced with borderline or atypical findings that require a nuanced and integrative approach beyond a single H&E-level assessment.

Beyond histopathological assessment, recognition of histological artefacts is critical to avoid overdiagnosis. Features induced by preoperative interventions—such as fine-needle aspiration or ethanol injection—can mimic true capsular or vascular invasion. Crush artefacts, peliosis, and intrathyroidal growth patterns may also be misinterpreted as evidence of malignancy if not carefully contextualized. Similarly, parathyromatosis and nodular hyperplasia in secondary hyperparathyroidism can exhibit morphological changes that resemble carcinoma but represent reactive or iatrogenic phenomena.

These diagnostic uncertainties carry significant clinical consequences. A diagnosis of parathyroid carcinoma not only alters surgical management but also imposes life-long implications for the patient. These may include frequent surveillance imaging, repeated biochemical monitoring, and the psychological burden associated with fear of recurrence or metastasis. Overcalling a benign or indeterminate lesion as carcinoma may thus lead to overtreatment and undue anxiety. Conversely, underdiagnosing a parathyroid carcinoma may delay appropriate intervention, increasing the risk of recurrence or progression.

To navigate this complexity, a multidisciplinary, integrative diagnostic model is essential. Clinical, biochemical, perioperative, histological, immunohistochemical, and—where available—molecular data must all be considered in concert. In ambiguous cases, consultation with an experienced endocrine pathologist is highly recommended, and referral to specialized centers may be warranted when advanced diagnostic tools or expertise are not available locally.

Ultimately, the goal is to strike a careful balance: to identify truly malignant cases in need of aggressive management while avoiding the pitfalls of overdiagnosis that could burden patients with unnecessary interventions and long-term follow-up. As diagnostic tools and molecular insights continue to evolve, they will hopefully offer a more refined stratification of risk in these diagnostically challenging tumors.
